# Ependymoma‐like tumor with mesenchymal differentiation harboring *C11orf95*‐*NCOA1*/*2* or ‐*RELA* fusion: A hitherto unclassified tumor related to ependymoma

**DOI:** 10.1111/bpa.12943

**Published:** 2021-02-12

**Authors:** Ran Tomomasa, Yasuhito Arai, Reika Kawabata‐Iwakawa, Kohei Fukuoka, Yoshiko Nakano, Natsuko Hama, Satoshi Nakata, Nozomi Suzuki, Yukitomo Ishi, Shinya Tanaka, Jun A. Takahashi, Yoshiaki Yuba, Mitsutaka Shiota, Atsushi Natsume, Michihiro Kurimoto, Yoshiki Shiba, Mikiko Aoki, Kazuki Nabeshima, Toshiyuki Enomoto, Tooru Inoue, Junya Fujimura, Akihide Kondo, Takashi Yao, Naoki Okura, Takanori Hirose, Atsushi Sasaki, Masahiko Nishiyama, Koichi Ichimura, Tatsuhiro Shibata, Junko Hirato, Hideaki Yokoo, Sumihito Nobusawa

**Affiliations:** ^1^ Department of Human Pathology Gunma University Graduate School of Medicine Maebashi Japan; ^2^ Division of Cancer Genomics National Cancer Center Research Institute Tokyo Japan; ^3^ Division of Integrated Oncology Research Gunma University Initiative for Advanced Research Maebashi Japan; ^4^ Department of Hematology/Oncology Saitama Children's Medical Center Saitama Japan; ^5^ Division of Brain Tumor Translational Research National Cancer Center Research Institute Tokyo Japan; ^6^ Department of Oncology Johns Hopkins University School of Medicine Baltimore MD USA; ^7^ Department of Neurosurgery Kitami Red Cross Hospital Kitami Japan; ^8^ Department of Neurosurgery Faculty of Medicine Hokkaido University Sapporo Japan; ^9^ Department of Cancer Pathology Faculty of Medicine, and WPI‐ICReDD Hokkaido University Sapporo Japan; ^10^ Department of Rehabilitation Medicine Rakusai Shimizu Hospital Kyoto Japan; ^11^ Department of Pathology Kitano Hospital the Tazuke Kofukai Medical Research Institute Osaka Japan; ^12^ Department of Pediatrics Kitano Hospital the Tazuke Kofukai Medical Research Institute Osaka Japan; ^13^ Department of Neurosurgery Nagoya University School of Medicine Nagoya Japan; ^14^ Department of Pathology Faculty of Medicine Fukuoka University Fukuoka Japan; ^15^ Department of Neurosurgery Faculty of Medicine Fukuoka University Fukuoka Japan; ^16^ Department of Pediatrics and Adolescent Medicine Juntendo University School of Medicine Tokyo Japan; ^17^ Department of Neurosurgery Juntendo University School of Medicine Tokyo Japan; ^18^ Department of Human Pathology Juntendo University School of Medicine Tokyo Japan; ^19^ Department of Radiology School of Medicine International University of Health and Welfare Narita Japan; ^20^ Pathology for Regional Communication Kobe University School of Medicine Kobe Japan; ^21^ Department of Diagnostic Pathology Hyogo Cancer Center Akashi Japan; ^22^ Department of Pathology Saitama Medical University School of Medicine Moroyama Japan; ^23^ Higashi Sapporo Hospital Sapporo Japan; ^24^ Gunma University Maebashi Gunma Japan; ^25^ Department of Pathology Public Tomioka General Hospital Tomioka Japan

**Keywords:** *C11orf95*, ependymoma, *NCOA1*, *NCOA2*, *RELA*

## Abstract

Recurrent fusion genes involving *C11orf95*, *C11orf95*‐*RELA*, have been identified only in supratentorial ependymomas among primary CNS tumors. Here, we report hitherto histopathologically unclassifiable high‐grade tumors, under the tentative label of “ependymoma‐like tumors with mesenchymal differentiation (ELTMDs),” harboring *C11orf95*‐*NCOA1*/*2* or ‐*RELA* fusion. We examined the clinicopathological and molecular features in five cases of ELTMDs. Except for one adult case (50 years old), all cases were in children ranging from 1 to 2.5 years old. All patients presented with a mass lesion in the cerebral hemisphere. Histologically, all cases demonstrated a similar histology with a mixture of components. The major components were embryonal‐appearing components forming well‐delineated tumor cell nests composed of small uniform cells with high proliferative activity, and spindle‐cell mesenchymal components with a low‐ to high‐grade sarcoma‐like appearance. The embryonal‐appearing components exhibited minimal ependymal differentiation including a characteristic EMA positivity and tubular structures, but histologically did not fit with ependymoma because they lacked perivascular pseudorosettes, a histological hallmark of ependymoma, formed well‐delineated nests, and had diffuse and strong staining for CAM5.2. Molecular analysis identified *C11orf95*‐*NCOA1*, ‐*NCOA2*, and ‐*RELA* in two, one, and two cases, respectively. t‐distributed stochastic neighbor embedding analysis of DNA methylation data from two cases with *C11orf95*‐*NCOA1* or ‐*NCOA2* and a reference set of 380 CNS tumors revealed that these two cases were clustered together and were distinct from all subgroups of ependymomas. In conclusion, although ELTMDs exhibited morphological and genetic associations with supratentorial ependymoma with *C11orf95*‐*RELA*, they cannot be regarded as ependymoma. Further analyses of more cases are needed to clarify their differences and similarities.

## Graphical abstract

- We report hitherto histopathologically unclassifiable high‐grade tumors, ELTMDs.
- ELTMDs exhibited minimal ependymal differentiation and C11orf95‐NCOA1/2 or ‐RELA.

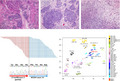

## 1 INTRODUCTION

Ependymomas develop anywhere throughout the central nervous system (CNS) and in all age groups, but are most commonly infratentorial in children and young adults, accounting for approximately 10% of pediatric intracranial brain tumors (1). Although ependymomas from different anatomical locations or from different age groups are hardly histopathologically distinguishable and the World Health Organization (WHO) grading is sometimes challenging because of not well defined criteria, recent genomic studies subdivided supratentorial (ST), posterior fossa, and spinal ependymomas into clinically meaningful and molecularly distinct subgroups, including ST ependymomas with *C11orf95*‐*RELA* (2, 3, 4, 5, 6, 7).

In the revised 4th edition of the WHO classification, ST ependymomas with *C11orf95*‐*RELA* are defined as a separate entity (8). In a large cohort study, ST ependymomas with *C11orf95*‐*RELA* accounted for 70% of all ST ependymomas, mostly in children; however, a significant portion (24%) was found in adults (3). Parker et al reported that the fusion genes resulted from clustered genomic rearrangements occurring in localized genomic regions, known as chromothripsis, at chromosome 11q12.1–11q13.3, and that the fusion proteins led to NF‐κB pathway activation with nuclear accumulation of p65/RelA (4). In addition, L1CAM, which was originally identified as a neural adhesion molecule essential for axonogenesis (9), was reported to be overexpressed in ST ependymomas with *C11orf95*‐*RELA* (4, 10, 11), suggesting that L1CAM is a target of aberrant signaling of the fusion proteins (4). Overexpression of both p65/RelA and L1CAM is identifiable by immunohistochemistry (4, 10, 11). Histologically, ST ependymomas with *C11orf95*‐*RELA* often exhibit clear cell morphology and branching vessels (8).

In ST ependymomas, *C11orf95* is also the fusion partner of other rare fusions with genes encoding transcription factors, such as *NCOA1*, *YAP1*, and *MAML2*, each with only one or two cases reported (4, 5, 12). The case of ependymoma with *C11orf95*‐*NCOA1* presented clear cell morphology and was diagnosed as anaplastic ependymoma (5); however, histology was not detailed for the other cases (4). Other than in ST ependymomas, as recurrent fusion genes involving *C11orf95*, only *C11orf95*‐*MKL2* were identified in chondroid lipomas, benign lipogenic tumors developing mainly in the extremities and limb girdles of adults (13, 14). Although it has been suggested that the zinc finger domains of C11orf95 may be essential oncogenic elements of these fusions involving *C11orf95*, the physiological function of C11orf95 is unknown (4). The breakpoints in *C11orf95* for *C11orf95*‐*RELA* in ST ependymomas are mostly between exons 2 and 3, whereas those for *C11orf95*‐*MKL2* in chondroid lipomas are within exon 5 (4, 5, 13, 14)

In this study, we report five cases of hitherto histopathologically unclassifiable high‐grade tumors with fusion genes involving *C11orf95*, with *NCOA1*, *NCOA2*, or *RELA* as fusion partners. These tumors, herein, referred to as “ependymoma‐like tumors with mesenchymal differentiation (ELTMDs),” demonstrated a similar histology, including small round blue cell components with minimal ependymal differentiation, but they cannot be regarded as embryonal tumors or ependymoma as a whole.

## 2 MATERIALS AND METHODS

### 2.1 Tumor samples

We searched the consultation archives of two authors (S. Nobusawa and J.H.), comprising approximately 2500 cases of brain tumors, for cases demonstrating a similar histology described below (for details see “RESULTS”), and found five such cases (Table 1). Sections for histological and genetic analyses were prepared from formalin‐fixed paraffin‐embedded (FFPE) tissue specimens. This study was conducted in accordance with the ethical committees of Gunma University and the National Cancer Center.

**TABLE 1:** Case list with clinical features and molecular status

Case	Age/Sex	Initial symptoms	Location	Size	Neuroimaging	Surgery	Radiation	Chemotherapy	Outcome	Molecular status
1	50/F	Headache, hemiplegia	Cerebrum (right frontal lobe)	4.5 cm	Cystic/solid, enhanced, calcification	GTR	Local 46 Gy	Temozolomide	RD (4 months)	*C11orf95*‐*RELA* ^a^
2	2.5/F	Ataxia, claudication	Cerebrum (right lateral ventricle)	5.5 cm	Cystic/solid, enhanced	PR	No	Multiagent*1 (1st and 2nd courses)	SD (3 months)	*C11orf95* (exon 5, partial)‐*NCOA1* (exon 15)^a^, ^b^, ^c^
2 Rec	–	–	–	–	Cystic/solid, enhanced	GTR	Local 27 Gy + CSI 23.4 Gy	Multiagent*1, (3rd and 4th courses), PBSCT	RD (2.8 years) → DOD (3.5 years)	–
3	1/M	Vomit, seizure	Cerebrum (left parietal lobe)	8 cm	Cystic/solid, enhanced, meningeal dissemination	GTR	No	Carboplatin, etoposide (at recurrence)	RD (1 years) → DOD (2.2 years)	*C11orf95* (exon 5, partial)‐*NCOA2* (exon 14)^a^, ^b^, ^c^ No matching methylation classes with calibrated score ≥0.9^d^ Methylation class ependymoma, RELA fusion with a low calibrated score (0.65)^d^
4	2/F	Seizure	Cerebrum (right frontal lobe)	3 cm	Solid, enhanced, well‐circumscribed, calcification	GTR	No	Multiagent*2 (BBSFOP)	NED (4.5 years)	*C11orf95*‐*RELA* ^a^
5	1.5/F	Seizure, strabismus	Cerebrum (left frontal lobe)	6 cm	Cystic/solid, enhanced	GTR	Local (proton) 50.4 Gy/30 Fr	Multiagent*3, PBSCT	RD → NED (3 years)	*C11orf95* (exon 5, partial)*‐NCOA1* (exon 14)^a^, ^b^, ^c^ No matching methylation classes with calibrated score ≥ 0.3^d^

Note

*1: etoposide, cyclophosphamide, cisplatin, vincristine, and intrathecal methotrexate and doxorubicin, followed by thiotepa and melphalan; *2: carboplatin, procarbazine, etoposide, cisplatin, vincristine, and , and cyclophosphamide; *3: vincristine, triple‐intrathecal (Methotrexate/Hydrocortisone/Cytarabine), followed by ifosfamide, cisplatin, and etoposide, and HIT 2000 protocol comprising cyclophosphamide, vincristine, methotrexate, carboplatin, and etoposide, and concomitant intrathecal methotrexate.

Abbreviations: CSI, craniospinal irradiation; DOD, died of disease; F, female; GTR, gross total resection; M, male; NED, no evidence of disease; PBSCT; peripheral blood stem cell transplantation; PR, partial resection; RD, recurrence of disease; SD, stable disease.

^a^ Identified by FISH analysis.

^b^ Identified by RNA sequencing

^c^ Identified by WES.

^d^ Analyzed by the DKFZ methylation classifier.

### 2.2 Conventional histological analysis

Three‐micrometer‐thick tissue sections were cut and stained with hematoxylin‐eosin or periodic acid–methenamine silver (PAM). Immunohistochemical staining was performed on FFPE tissue sections. Primary antibodies against the following antigens were applied: vimentin (V9; 1:200; Dako, Glostrup, Denmark), glial fibrillary acidic protein (GFAP) (1:5000) (15), Olig2 (1:5000) (16), cytokeratin (CAM5.2; 1:5; BD Bioscience, San Jose, CA, USA), α‐smooth muscle actin (αSMA) (1A4; 1:3200; BioMakor, Rehovot, Israel), epithelial membrane antigen (EMA) (E29; 1:100; Dako), synaptophysin (27G12; 1:200; Novocastra, Newcastle upon Tyne, UK), NeuN (A60; 1:1000; Chemicon, Temecula, CA, USA), podoplanin (D2‐40; prediluted; Nichirei, Tokyo, Japan), CD99 (12E7; 1:50; Dako), L1CAM (UJ127; 1:100; Novus Biologicals, Littleton, CO, USA), p65/RelA (D14E12; 1:400; Cell Signaling Technology, Danvers, MA, USA), BAF47/INI1 (BAF47; 1:100; BD Bioscience, San Jose, CA, USA), BRG1 (polyclonal; 1:1000; Millipore, Temecula, CA, USA), and Ki‐67 (MIB‐1; 1:100; Dako). For coloration, a commercially available biotin‐streptavidin immunoperoxidase kit (Histofine, Nichirei) and diaminobenzidine were employed.

### 2.3 RNA sequencing and reverse transcriptase‐polymerase chain reaction (RT‐PCR)

Total RNA was extracted from FFPE (cases 2 and 5) or frozen (case 3) samples. RNA sequencing and RT‐PCR were performed as described in Supporting Information.

### 2.4 Fluorescence in situ hybridization (FISH) analysis

Dual‐probe hybridization using an intermittent microwave irradiation method was employed using 4‐μm‐thick FFPE tissue sections, as described previously (17). Probes for *C11orf95*, *RELA*, *NCOA1*, and *NCOA2* were prepared from bacterial artificial chromosome (BAC) clones, as described previously (Table S1) (18, 19). The BAC clones were labeled with either ENZO Orange‐dUTP or ENZO Green‐dUTP (Abbott Molecular Inc., Des Plaines, IL, USA), and metaphase FISH to verify clone mapping positions was performed using the peripheral blood cell cultures of a healthy donor.

### 2.5 Whole exome sequencing (WES)

WES was performed on DNA isolated from FFPE tissue of cases 2–5 with sufficient quality and quantity using a NextSeq 500 DNA sequencer as described in Supporting Information. Selected variants observed in more than two cases were categorized as follows: COSMIC database (https://cancer.sanger.ac.uk/cosmic)‐registered variants, truncation mutation (not registered in COSMIC database), or variants of unknown significance (VUS) (Table S2).

### 2.6 Genome‐wide DNA methylation analysis

DNA of sufficient quality and quantity was extracted from cases 3 (frozen sample) and 5 (FFPE sample), and bisulfite modification of DNA was performed using an EZ Methylation DNA Kit (Zymo Research, CA, USA). Methylation profiling was performed as in Supporting Information.

### 2.7 Array comparative genomic hybridization (CGH)

DNA from cases 2–5 of sufficient quality and quantity extracted from FFPE samples was analyzed by array CGH as described in Supporting Information.

## 3 RESULTS

### 3.1 Clinical findings

**FIGURE 1:**
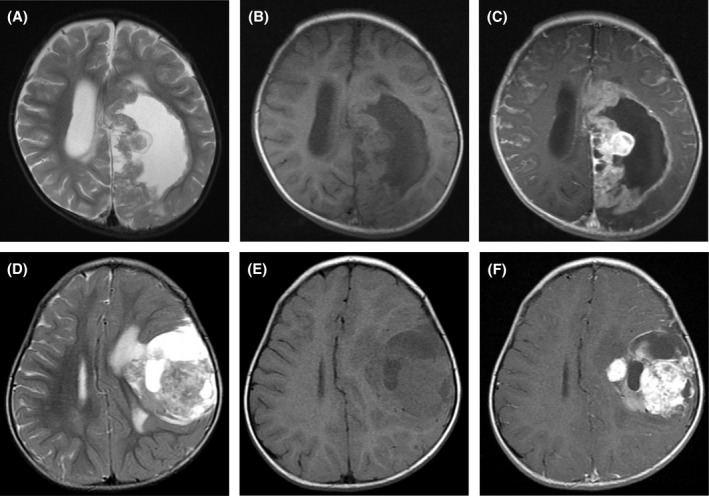
Radiological images of representative cases; (A–C) case 3, (D–F): case 5. Both tumors consist of a cystic component and solid component. The solid components exhibit iso‐ to high intensity in the cerebral cortex on T2‐weighted images (A and D) and iso‐intensity on T1‐weighted images (B and E), and were heterogeneously enhanced after gadolinium injection (C and F). Meningeal enhancement surrounding the surface of the brain and spinal cord is observed, suggesting meningeal dissemination (C)

Relevant clinical data are summarized in Table 1. Case 1 was in an adult (50 years old), and cases 2–5 were in children ranging from 1 to 2.5 years old. All patients presented with a mass lesion in the cerebral hemisphere; cases 1, 4, and 5 were located in the superficial portion, and the others were in the deep portion involving the lateral ventricle. Tumors demonstrated iso‐ to high intensity on T2‐weighted images (Figure 1A,D), iso‐intensity on T1‐weighted images (Figure 1B,E), and were heterogeneously enhanced after gadolinium injection (Figure 1C,F). Cases 1–3, and 5 possessed cystic components, and cases 1 and 4 with available computed tomography images had calcification. Patients in cases 1, and 3–5 underwent primary gross‐total resection, whereas that in case 2 underwent two‐staged resection over 3 months. Of four patients with a follow‐up period longer than 2 years, those in cases 2 and 3 died of the disease (3.5 and 2.2 years, respectively), and those in cases 4 and 5 were alive without evidence of disease at 4.5 and 3.5 years after initial surgery, respectively.

### 3.2 Histopathological findings

**FIGURE 2:**
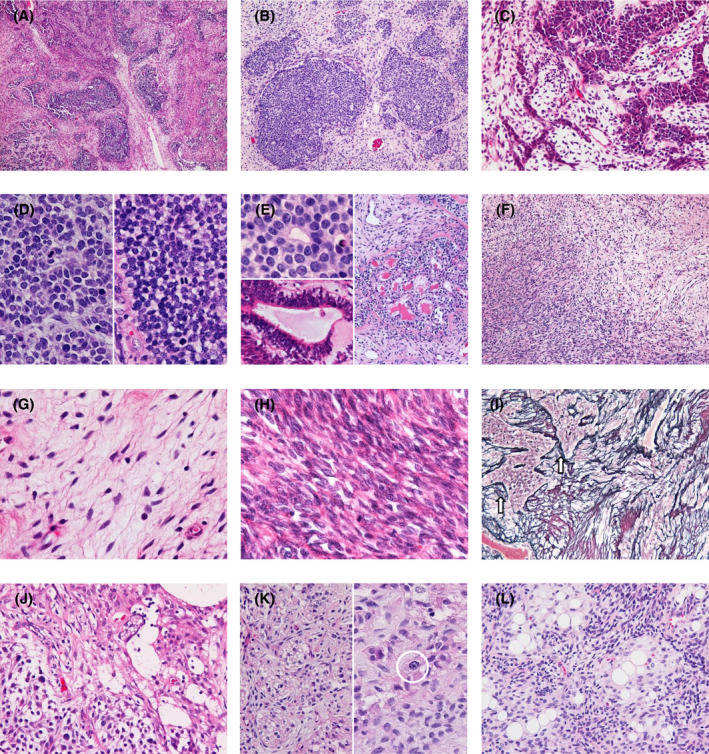
Microscopic appearance of the ependymoma‐like tumors with mesenchymal differentiation (ELTMDs). (A) The tumor is mainly composed of embryonal‐appearing components and spindle‐cell mesenchymal components (case 1). (B–E) Microscopic appearance of the embryonal‐appearing components. Variably sized and shaped tumor cell nests separated by the mesenchymal components (B, case 5). Tumor cell nests (right), thin cord‐like structures, and minute small clusters (left) (C, case 1). The components exhibit a highly cellular, poorly differentiated, hyperchromatic, and mitotically active histological appearance composed of small tumor cells with scant cytoplasm (D; left, case 2; right, case 5). Small to large tubular structures, with some containing eosinophilic amorphous material (E; top left, case 5; bottom left, case 1; right, case 4). (F–H) Microscopic appearance of the mesenchymal components. A transition between low‐ (top right) to high‐grade sarcoma‐like (bottom left) histologies (F, case 4). The low‐grade area includes mitotically indolent spindle tumor cells with low cellularity (G, case 3). The high‐grade sarcoma‐like area exhibits the dense proliferation of spindle cells with larger nuclei and high mitotic activity (H, case 1). (I) Periodic acid–methenamine silver staining exhibits abundant pericellular reticulin in the mesenchymal components. The arrows indicate the embryonal‐appearing components (case 5). (J and K) Microscopic appearance of the glioneuronal components. Astrocyte‐like tumor cells in top right and neurocyte‐like tumor cells in bottom left (J, same area as Figure 3F,G, case 5). Acinus‐like structures with the mesenchymal component trapping the glioneuronal tumor cells (K, left, case 2). The circle indicates a ganglioid tumor cell (K, right, case 2). Lipomatous metaplasia found is limited (L, case 4). Original magnification: A x40; B, E right, F x100; C, E bottom left, I, J, K left, L x200; D, G, H, K right x400; E top left x600

All five cases demonstrated a mixed histology; the major components observed in all cases were embryonal‐appearing components and spindle‐cell mesenchymal components (Figure 2A). The embryonal‐appearing components were characterized by variably sized and shaped tumor cell nests separated mostly by the mesenchymal components (Figure 2B). Thin cord‐like structures and minute small clusters were also observed (Figure 2C). The components exhibited a highly cellular, poorly differentiated, hyperchromatic, and mitotically active histological appearance composed of small tumor cells with scant cytoplasm (Figure 2D). Small to large tubular structures were found in limited parts of the components, with some containing eosinophilic amorphous material (Figure 2E). The mesenchymal components were composed of relatively monotonous spindle cells in a fascicular or diffuse pattern, ranging from low‐ to high‐grade sarcoma‐like histologies (Figure 2F); the former exhibited mitotically indolent tumor cells with low cellularity in a collagenous, edematous, or myxoid background (Figure 2G), whereas the latter demonstrated a dense proliferation of spindle cells with larger nuclei and higher mitotic activity (Figure 2H). PAM staining revealed abundant pericellular reticulin in the mesenchymal components (Figure 2I). The third element was glioneuronal components, observed in cases 2, 3, and 5, consisting of astrocyte‐like tumor cells with oval nuclei and eosinophilic cytoplasm with processes and neurocyte‐like tumor cells with round nuclei and clear cytoplasm (Figure 2J). A small number of ganglioid tumor cells with relatively large nuclei with prominent nucleoli were also observed (Figure 2K). The components occasionally assumed acinus‐like structures with the mesenchymal component trapping the glioneuronal tumor cells (Figure 2K). Mitoses were rare in the glioneuronal components. A small area with lipomatous metaplasia was found only in case 4 (Figure 2L).

**FIGURE 3:**
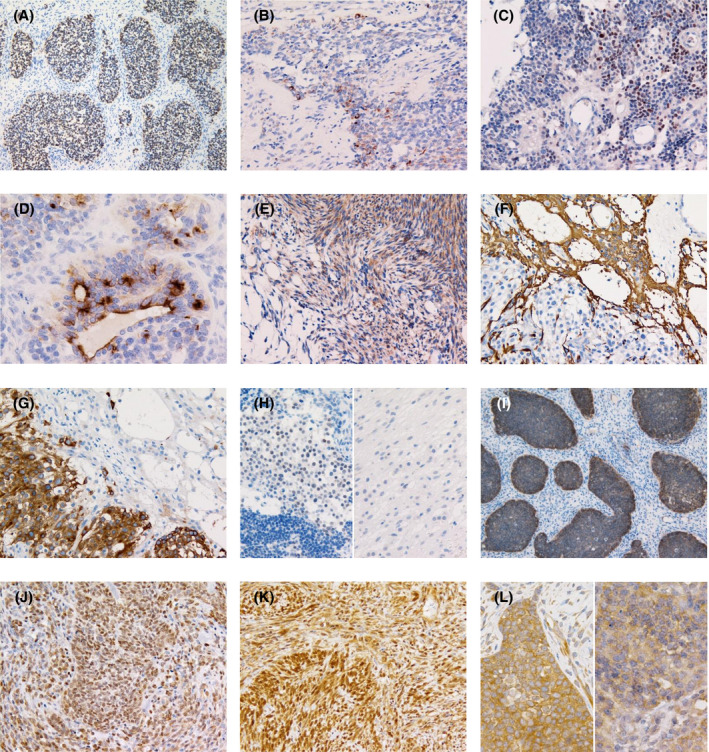
Immunohistochemistry of ELTMDs. (A) Tumor cells of the embryonal‐appearing components are diffusely positive for CAM5.2 (case 5). Focal GFAP (B, case 4) and Olig2 (C, case 3) immunoreactivity in the embryonal‐appearing components. (D) EMA staining exhibits a dot‐like pattern of cytoplasmic positivity and linear positivity along the apical surface of the tubular structures in the embryonal‐appearing components (case 2). (E) The mesenchymal components are positive for vimentin (case 4). GFAP (F) and synaptophysin (G) expression is observed in the astrocyte‐ and neurocyte‐like tumor cells of the glioneuronal components, respectively (F,G, same area as Figure 2J, case 5). (H). Weak NeuN expression is observed in the neurocyte‐like tumor cells (left), whereas the embryonal‐appearing components (bottom in left) and astrocyte‐like tumor cells are negative (right). (I). L1CAM expression is observed in the embryonal‐appearing components (case 5). Diffuse nuclear staining of p65/RelA (J, case 1; K case 4). (L). Although cytoplasmic p65/RelA immunoreactivity is seen, nuclear staining is absent (left, case 5; right, case 3). Original magnification: A, I x100; B, C, E‐H, J, K x200; D, L x400

On immunohistochemistry, tumor cells of the embryonal‐appearing components were diffusely positive for CAM5.2 (Figure 3A). GFAP and Olig2 immunoreactivity in the components was focally identified only in cases 4 and 3, respectively (Figure 3B,C). EMA staining exhibited a dot‐like pattern of cytoplasmic positivity and linear positivity along the apical surface of some of the tubular structures in the embryonal‐appearing components (Figure 3D). The mesenchymal components were positive for vimentin in all five cases (Figure 3E) and αSMA was negative in the two cases tested (cases 3 and 4). A limited number of spindle tumor cells was positive for GFAP and podoplanin in all cases. In the glioneuronal components, the astrocyte‐like tumor cells were positive for GFAP and the neurocyte‐like tumor cells were positive for synaptophysin (Figure 3F,G). The neurocyte‐like tumor cells were weakly positive for NeuN (Figure H). Reactivity for Olig2 was observed in the astrocyte‐like tumor cells to varying degrees. CD99 was negative in all cases. L1CAM expression was almost exclusively found in the embryonal‐appearing components in all cases (Figure 3I). Nuclear accumulation of p65/RelA was detected in cases 1 and 4, but not in cases 2, 3, or 5 (Figure 3J–L). Nuclear expression of INI1 and BRG1 was retained throughout the tumor tissue in all cases. MIB‐1 labeling indices were high in the embryonal‐appearing components and high‐grade mesenchymal components, with the highest ranging from 30% to 57%.

**FIGURE 4:**
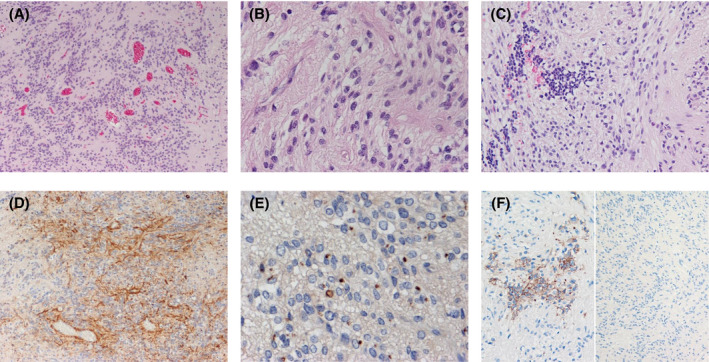
Histological and immunohistochemical findings of the recurrent tumor in case 2. (A and B) The ependymoma‐like histology composing most of the tumor. Perivascular pseudorosettes with anuclear zones are observed. (C). A limited amount of the embryonal‐appearing components showing small tumor cell nests in the ependymoma‐like components. (D) The ependymoma‐like tumor cells are immunoreactive for GFAP, with perivascular cytoplasmic processes exhibiting particularly strong staining. (E). Dot‐like and ring‐like patterns of cytoplasmic EMA positivity. (F) L1CAM expression is found in the embryonal‐appearing components (left) but not in the ependymoma‐like components (right). Original magnification: A, D, F right x100; C, F left x200; B, E x400

Most of the specimen from the second operation in case 2, besides the components described above, displayed an ependymoma‐like histology, that is, the proliferation of tumor cells with round to ovoid nuclei and eosinophilic cytoplasmic processes, exhibiting perivascular pseudorosettes with anuclear zones (Figure 4A–C). One mitosis was detected in 10 high‐power fields in this element. The ependymoma‐like tumor cells were immunoreactive for GFAP, with perivascular cytoplasmic processes having particularly strong staining (Figure 4D). Dot‐like and ring‐like patterns of cytoplasmic EMA positivity were observed in this component (Figure 4E). The components were negative for CAM5.2 staining. L1CAM expression was limited in the embryonal‐appearing components (Figure 4F). Nuclear accumulation of p65/RelA was not detected. MIB‐1 labeling index was 3% in the ependymoma‐like components.

### 3.3 Genetic analysis

**FIGURE 5:**
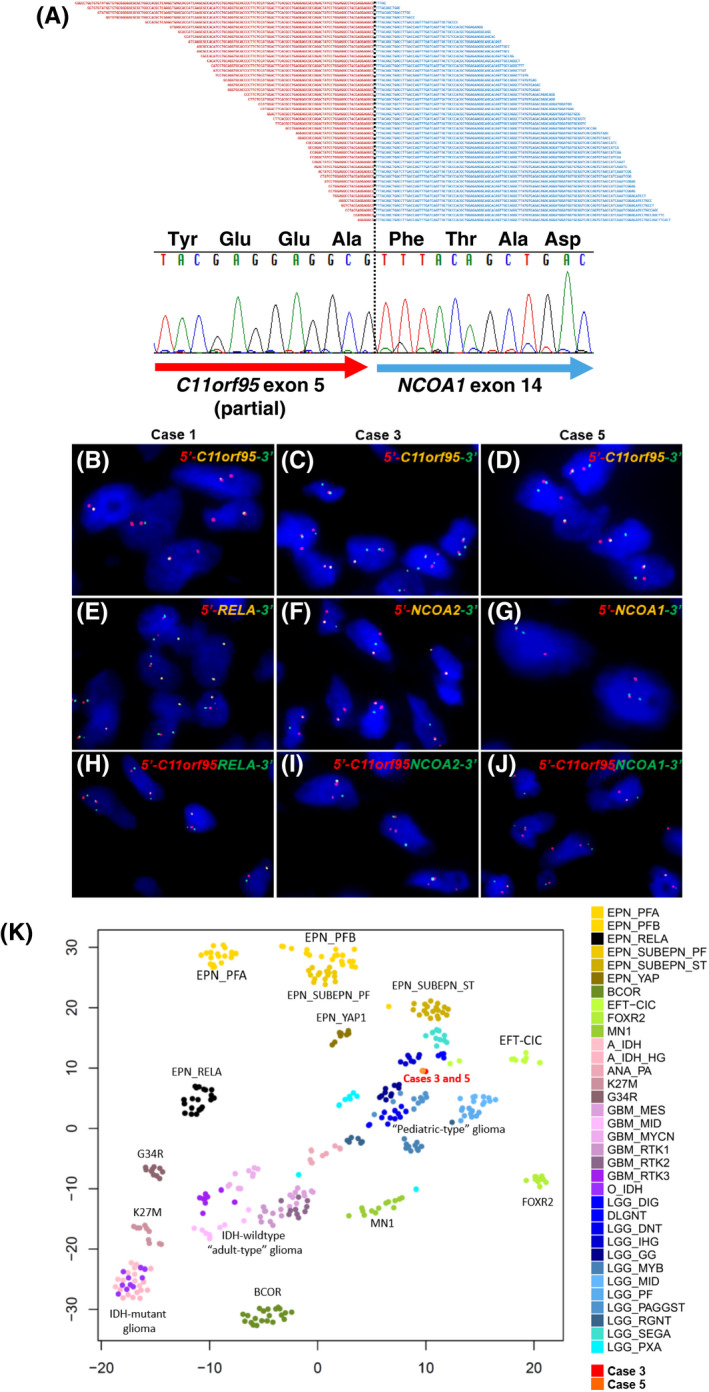
(A) *C11orf95*‐*NCOA1* fusion in case 5 was identified by target RNA sequencing, and confirmed by RT‐PCR and Sanger sequencing. Reference sequence: *C11orf95*, NM_001144936; *NCOA1*, NM_003743. (B–J) Representative fluorescence in situ hybridization results. (B–G) Rearrangement with fused (normal) and break‐apart signals in each case. (B–D) 5′‐*C11orf95*, orange signal; *C11orf95*‐3′, green signal. In case 1, one fused signal and one isolated orange signal are observed, and isolated green signals are lost (B). (E) 5′‐*RELA*, orange signal; *RELA*‐3′, green signal. (F) 5′‐*NCOA2*, orange signal; *NCOA2*‐3′, green signal. (G) 5′‐*NCOA1*, orange signal; *NCOA1*‐3′, green signal. (H–J) Fusion signals in each case: 5′‐*C11orf95*‐*RELA*‐3′ (H), 5′‐*C11orf95*‐*NCOA2*‐3′ (I), and 5′‐*C11orf95*‐*NCOA1*‐3′ (J). (K) t‐distributed stochastic neighbor embedding analysis of DNA methylation data from cases 3 and 5 and a reference set of 380 CNS tumors. Reference methylation classes: EPN_PFA, ependymoma, posterior fossa group A; EPN_PFB, ependymoma, posterior fossa group B; EPN_RELA, ependymoma, RELA fusion; EPN_SUBEPN_PF, subependymoma, posterior fossa; EPN_SUBEPN_ST, subependymoma, supratentorial; EPN_YAP, ependymoma, YAP fusion; BCOR, high‐grade neuroepithelial tumor (HGNET) with BCOR alteration; EFT‐CIC, Ewing sarcoma family tumor with CIC alteration; FOXR2, neuroblastoma with FOXR2 activation; MN1, HGNET with MN1 alteration; A_IDH(_HG), (high‐grade) astrocytoma, IDH‐mutation; ANA_PA, anaplastic pilocytic astrocytoma; K27M, diffuse midline glioma H3 K27M‐mutant; G34R, glioblastoma, H3.3 G34‐mutant; GBM_MES, glioblastoma, IDH‐wildtype, subclass mesenchymal; GBM_MID, glioblastoma, IDH‐wildtype, subclass midline; GBM_MYCN, glioblastoma, IDH‐wildtype, subclass MYCN; GBM_RTK1‐3, glioblastoma, IDH‐wildtype, subclass RTK I‐III; O_IDH, oligodendroglioma, IDH‐mutant and 1p/19q‐codeleted; LGG_DIG, desmoplastic infantile ganglioglioma; DLGNT, diffuse leptomeningeal glioneuronal tumor; LGG_DNT, dysembryoplastic neuroepithelial tumor; LGG_IHG, infantile hemispheric glioma LGG_GG, ganglioglioma; LGG_MYB, low‐grade glioma with MYB/MYBL1 rearrangement, LGG_MID, midline pilocytic astrocytoma; LGG_PF, posterior fossa pilocytic astrocytoma, LGG_PAGGST, supratentorial/hemispheric pilocytic astrocytoma/ganglioglioma, LGG_RGNT, rosette‐forming glioneuronal tumor; LGG_SEGA, subependymal giant cell astrocytoma; LGG_PXA, pleomorphic xanthoastrocytoma

RNA sequencing identified in‐frame fusions of *C11orf95* (exon 5) and *NCOA1* (exon 15), *C11orf95* (exon 5) and *NCOA2* (exon 14), and *C11orf95* (exon 5) and *NCOA1* (exon 14) in cases 2, 3, and 5, respectively (Figure 5A, Table 1, and Figure S1). The breakpoints in *C11orf95* for these fusions were within exon 5. The fusion in case 5 was confirmed by RT‐PCR and Sanger sequencing (Figure 5A).

FISH analysis using break‐apart *C11orf95* probes revealed positive signals of *C11orf95* rearrangement in all five cases (Figure 5B–D). In cases 1 and 4, break‐apart signals of *RELA* and fusion signals of *C11orf95*‐*RELA* were observed (Figure 5E,H, Table 1). In the remaining cases, break‐apart signals of *NCOA1* (cases 2 and 5) or *NCOA2* (case 3) and fusion signals of *C11orf95*‐*NCOA1* (cases 2 and 5) or *C11orf95*‐*NCOA2* (case 3) were observed (Figure 5F,G,I,J and Table 1).

Based on analysis of cases 2‐5 by WES, variants shared by more than two cases are listed in Table S2. No variants, including COSMIC database‐registered variants, were assigned as pathogenic in ClinVar (https://www.ncbi.nlm.nih.gov/clinvar/) and we did not observe any obvious oncogenic variants. *C11orf95*‐*NCOA1*/2 detected by RNA sequencing in cases 2, 3, and 5 were also identified by WES (Figure S2), whereas *C11orf95*‐*RELA* detected by FISH analysis in case 4 was not identified, possibly because neither of the breakpoints in *C11orf95* or *RELA* is within or near an exon.

By methylation analysis using the DKFZ methylation classifier, case 3 was classified as no matching methylation classes with a confidence threshold of the calibrated score ≥0.9, and as methylation class ependymoma, RELA fusion with a low calibrated score (0.65) (Table 1). Case 5 was classified as no matching methylation classes with a calibrated score ≥0.3 (Table 1). t‐distributed stochastic neighbor embedding analysis of DNA methylation data from cases 3 and 5 and a reference set of 380 CNS tumors demonstrated that cases 3 and 5 were clustered together and distinct from all subgroups of ependymomas (Figure 5K).

By array CGH, no apparent copy number changes other than small deletions and gains in regions of known benign copy number variants (polymorphisms) reported in the Database of Genomic Variants (DGV) (http://dgv.tcag.ca/dgv/app/home) were found in cases 2‐5 (Figure S3A). Copy number analysis using the DKFZ methylation classifier also demonstrated stable chromosomal status with no apparent copy number changes in cases 3 and 5 (Figure S3B).

## 4 DISCUSSION

In this report, we described five high‐grade CNS tumors exhibiting distinct histopathological and molecular features, analyzed them as a group of tumors under the tentative label of ELTMD, and demonstrated that fusion genes involving *C11orf95* are not restricted to histologically defined ependymomas.

The tumors collected for this study displayed a mixed histology, and one of the major components demonstrated embryonal‐appearing histology (Figure 2B–D), being the most similar to anaplastic ependymoma considering the minimal ependymal differentiation observed in the components and the detected fusion genes, *C11orf95*‐*NCOA1*/*2* or ‐*RELA*. Histopathological features of ependymal differentiation in the embryonal‐appearing components of ELTMDs include a dot‐like pattern of cytoplasmic EMA positivity and small to large tubular structures with linear EMA positivity along the apical surface resembling ependymal rosettes and tubules, which are histological characteristics of ependymoma (Figures 2E and 3D). Meanwhile, ependymal differentiation is not restricted to ependymoma, but is generally accepted in several other primary CNS tumors, including angiocentric glioma, astroblastoma, chordoid glioma, and papillary tumor of the pineal region [reviewed in reference (20)], and the former 3 are known to be associated with specific genetic alterations: *MYB*‐*QKI* fusion, *MN1* fusions, and *PRKCA* D463H mutation, respectively (21, 22, 23, 24, 25).

Despite the above‐mentioned ependymal differentiation and genetic associations, we think that the embryonal‐appearing components histologically did not fit with anaplastic ependymoma because of the following points. (1) Perivascular pseudorosettes with perivascular cytoplasmic processes exhibiting particularly strong GFAP staining can be found, almost by definition, in practically all (anaplastic) ependymomas (1, 26, 27); however, these formations were not present in the embryonal‐appearing components throughout the tumor tissues in all cases. (2) In (anaplastic) ependymoma, staining for CAM5.2 is focal at best, and diffuse and strong staining for CAM5.2, which was observed in the embryonal‐appearing components of all ELTMDs in the current study (Figure 4A), is not consistent with a diagnosis of (anaplastic) ependymoma (26, 28). In addition, the embryonal‐appearing components lacked clear cell morphology with branching vessels, histological features often observed in ST ependymomas with *C11orf95*‐*RELA* (8). Microvascular proliferation or palisading necrosis, findings indicative of malignancy in ependymoma (27), were not noted. From a genetic standpoint, ST ependymomas with *C11orf95*‐*RELA* were reported to typically have abundant copy number changes; frequent changes were focal losses and gains on chromosome11q (including chromothripsis), losses involving chromosomes 3, 9 (often resulting in homozygous deletion of *CDKN2A*), 10, and 22, and gain of chromosome 1q (3, 29). However, although only one case of ELTMD with *C11orf95*‐*RELA* was analyzed by array CGH and the status of copy number changes in the only one reported case of ependymoma with *C11orf95*‐*NCOA1* is unknown (5), all 4 ELTMDs in the current study displayed stable chromosomal profiles (Figure S3), which may be a difference from ST ependymomas with *C11orf95*‐*RELA* and must be further analyzed in more cases.

Ependymomas with sarcomatous changes are called ependymosarcomas, which are included in gliosarcoma, a variant of IDH‐wildtype glioblastoma, in the current WHO scheme of CNS tumors (30). The sarcomatous components were reported to be mainly composed of atypical spindle cells, and some cases had pleomorphic cells and heterologous (osseous, cartilaginous, and rhabdomyoblastic) differentiation in the components (31). The other major components in ELTMD are spindle‐cell mesenchymal components; therefore, considering the overall tumor composition, ELTMD is more similar to ependymosarcoma than to anaplastic ependymoma. We thought that some ELTMDs may have been regarded as ependymosarcoma; however, such cases were not found in the literature including the largest series with 11 cases (31), considering the histopathological description, including the presence of perivascular pseudorosettes and absent to focal and weak CAM5.2 staining in the ependymoma components. *C11orf95*‐*RELA* has not been examined in primary ependymosarcomas; however, *C11orf95*‐*RELA* was detected both in primary anaplastic ependymoma and recurrent sarcoma in a patient; the latter developed after chemotherapy and radiation (32).

In this study, we identified *C11orf95*‐*NCOA1/2* in three ELTMDs. Fusion genes involving *NCOA1*/*2* have been recurrently identified in several types of soft tissue tumors and acute leukemia (33, 34). In the CNS, the case of ST anaplastic ependymoma presenting clear cell morphology with *C11orf95*‐*NCOA1* was reported (5). Quite recently, Keenan et al reported three cases of “infratentorial” ependymomas with *C11orf95*‐*NCOA2*, ‐*MAML2*, or ‐*RELA* showing histological features closely resembling ST ependymomas with *C11orf95*‐*RELA* (35). DNA methylation analysis demonstrated that all these infratentorial ependymomas clustered together with ST ependymomas with *C11orf95*‐*RELA* (35). On the contrary, our study revealed that two ELTMDs with *C11orf95*‐*NCOA1/2* were epigenetically clearly distinct from ST ependymomas with *C11orf95*‐*RELA* (Figure 5K). Taken together, histologically defined ependymomas with *C11orf95* fusion including *C11orf95*‐*NCOA2* may be epigenetically different from ELTMDs with *C11orf95*‐*NCOA1/2*. Further DNA methylation analysis of ELTMDs, especially those with *C11orf95*‐*RELA*, is necessary to clarify the epigenetic relationships between ELTMDs with *C11orf95*‐*RELA* and ependymomas with *C11orf95*‐*RELA*, and between ELTMDs with *C11orf95*‐*RELA* and ELTMDs with *C11orf95*‐*NCOA1/2*.

By immunohistochemistry, although L1CAM was reported to be typically expressed in a diffuse and strong manner in ST ependymomas with *C11orf95*‐*RELA* (10, 11, 36, 37, 38), L1CAM expression was almost exclusively found in the embryonal‐appearing components in all cases of primary ELTMD and the recurrent tumor in case 2 regardless of the fusion partners of *C11orf95* (Figure 3I). The function of L1CAM may be required in the most proliferative components with ambiguous differentiation in ELTMDs, and its expression may be lost along with mesenchymal, glioneuronal, and ependymal (in the recurrent tumor of case 2) differentiation with lower proliferative activity. L1CAM expression, though in few cases, was also reported in ST ependymomas with *C11orf95*‐*YAP1* and *C11orf95*‐*MAML2* (4, 12). In ST ependymomas with *YAP1*‐*MAMLD1*, another molecular subgroup of ST ependymoma, no positivity for L1CAM was observed in any of the 11 cases tested (39). Together with our results, L1CAM expression may be more related to *C11orf95* than to *RELA* in ST ependymomas and ELTMDs with fusion genes involving *C11orf95*. On the contrary, nuclear accumulation of p65/RelA was detected only in cases with *C11orf95*‐*RELA* (cases 1 and 4), but not in cases with *C11orf95*‐*NCOA1*/*2* (cases 2, 3, or 5) in this study (Figure 3J–L), consistent with the fusion protein C11orf95‐RELA leading to NF‐κB pathway activation (4).

Although primary ELTMDs cannot be regarded as anaplastic ependymoma or ependymosarcoma, the recurrent tumor in case 2 predominantly displayed a classic low‐grade ependymoma histology, including perivascular pseudorosettes with an accentuated perivascular staining pattern of GFAP, and dot‐like and ring‐like patterns of cytoplasmic EMA positivity (Figure 4). Chemotherapy performed after the first surgery may be responsible for the morphological and phenotypical changes; however, this phenomenon may reflect the intrinsic ependymal nature of ELTMD.

In conclusion, although ELTMDs demonstrated minimal ependymal differentiation and genetic association with ST ependymoma with *C11orf95*‐*RELA*, they cannot be regarded as (anaplastic) ependymoma or ependymosarcoma by the current WHO classification. Given the small number of cases examined in the current study, further clinicopathological and genetic analyses of more cases are needed to clarify their differences and similarities, and the possibility of them being included in the spectrum of ependymoma by the more molecularly oriented definition of ependymoma in the future cannot be excluded.

## CONFLICT OF INTEREST

The authors declare no conflict of interest.

## AUTHOR CONTRIBUTIONS

Sumihito Nobusawa designed the study; Ran Tomomasa, Takanori Hirose, Atsushi Sasaki, Junko Hirato, and Sumihito Nobusawa performed the pathological analysis; Ran Tomomasa, Yasuhito Arai, Reika Kawabata‐Iwakawa, Kohei Fukuoka, Yoshiko Nakano, Natsuko Hama, Masahiko Nishiyama, Koichi Ichimura, Tatsuhiro Shibata, and Sumihito Nobusawa performed the laboratory research; cases and clinical data were provided by Nozomi Suzuki, Yukitomo Ishi, Shinya Tanaka, Jun A. Takahashi, Yoshiaki Yuba, Mitsutaka Shiota, Atsushi Natsume, Michihiro Kurimoto, Yoshiki Shiba, Mikiko Aoki, Kazuki Nabeshima, Toshiyuki Enomoto, Tooru Inoue, Junya Fujimura, Akihide Kondo, and Takashi Yao; Ran Tomomasa, Satoshi Nakata, Naoki Okura, and Sumihito Nobusawa analyzed and interpreted the data; Ran Tomomasa, Satoshi Nakata, and Sumihito Nobusawa wrote the manuscript; Junko Hirato and Hideaki Yokoo participated in construction of the manuscript and revised it critically; and all authors accepted the final version of the manuscript.

## Supporting information

**FIGURE S1***C11orf95*‐*NCOA1*/*2* fusions identified by target RNA sequencing in cases 2 and 3. Sequence reads spanning the breakpoints are illustrated. The breakpoint junctions contain 2‐ and 11‐bp insertions, respectively. Reference sequence: *C11orf95*, NM_001144936; *NCOA1*, NM_003743; *NCOA2*, NM_006540

**FIGURE S2** Identification of *C11orf95*‐*NCOA1*/*2* fusion events by whole exome sequencing. Fusions between exon 5 of *C11orf95* and introns 14 and 13 of *NCOA1* (cases 2 and 5, respectively), and intron 13 of *NCOA2* (case 3) are observed. Reads are sorted and colored based on the location of their mate reads: orange (cases 2 and 5) and purple (case 3), mate reads in chromosome 11 (*C11orf95*); brown, mate reads in chromosome 2 (*NCOA1*, cases 2 and 5) and in chromosome 8 (*NCOA2*, case 3)

**FIGURE S3** (A) In case 4 with *C11orf95*‐*RELA*, array comparative genomic hybridization shows no apparent copy number changes in chromosomes 1, 3, 9, 10, 11, or 22, where supratentorial ependymomas with *C11orf95*‐*RELA* were reported to have abundant copy number changes. (B) Copy number analysis using the DKFZ methylation classifier demonstrated stable chromosomal status with no apparent copy number changes in cases 3 and 5

Supplementary Material

**TABLE S1** Fluorescence in situ hybridization probes

**TABLE S2** Variants observed in more than two cases by whole exome sequencing

## Data Availability

Derived data supporting the findings of this study are available from the corresponding author on request.
