# Supplementary material for: Ependymoma‐like tumor with mesenchymal differentiation harboring C11orf95‐NCOA1/2 or ‐RELA fusion: A hitherto unclassified tumor related to ependymoma
Source: Brain Pathol. 2021 Feb 12;31(3):e12943. doi: 10.1111/bpa.12943 (PMC8412126; doi:10.1111/bpa.12943)
Supplement: Supplementary file 1 — FIGURE S1C11orf95‐NCOA1/2 fusions identified by target RNA sequencing in cases 2 and 3. Sequence reads spanning the breakpoints are illustrated. The breakpoint junctions contain 2‐ and 11‐bp insertions, respectively. Reference sequence: C11orf95, NM_001144936; NCOA1, NM_003743; NCOA2, NM_006540 [file BPA-31-e12943-s001.pptx]

## Slide 1
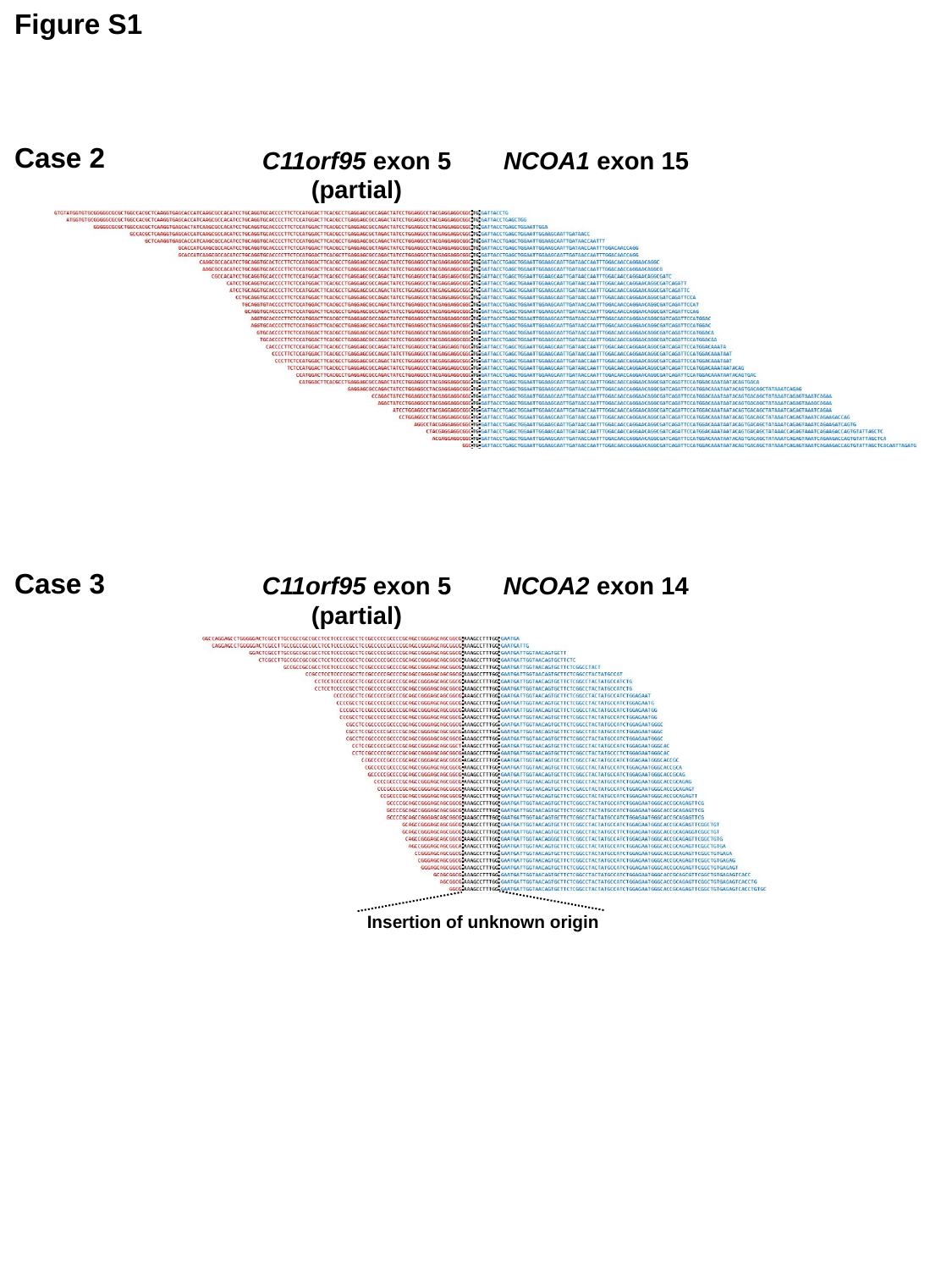

Figure S1
Case 2
NCOA1 exon 15
C11orf95 exon 5
(partial)
Case 3
NCOA2 exon 14
C11orf95 exon 5
(partial)
Insertion of unknown origin
