# Supplementary material for: Ependymoma‐like tumor with mesenchymal differentiation harboring C11orf95‐NCOA1/2 or ‐RELA fusion: A hitherto unclassified tumor related to ependymoma
Source: Brain Pathol. 2021 Feb 12;31(3):e12943. doi: 10.1111/bpa.12943 (PMC8412126; doi:10.1111/bpa.12943)
Supplement: Supplementary file 2 — FIGURE S2 Identification of C11orf95‐NCOA1/2 fusion events by whole exome sequencing. Fusions between exon 5 of C11orf95 and introns 14 and 13 of NCOA1 (cases 2 and 5, respectively), and intron 13 of NCOA2 (case 3) are observed. Reads are sorted and colored based on the location of their mate reads: orange (cases 2 and 5) and purple (case 3), mate reads in chromosome 11 (C11orf95); brown, mate reads in chromosome 2 (NCOA1, cases 2 and 5) and in chromosome 8 (NCOA2, case 3) [file BPA-31-e12943-s002.pptx]

## Slide 1
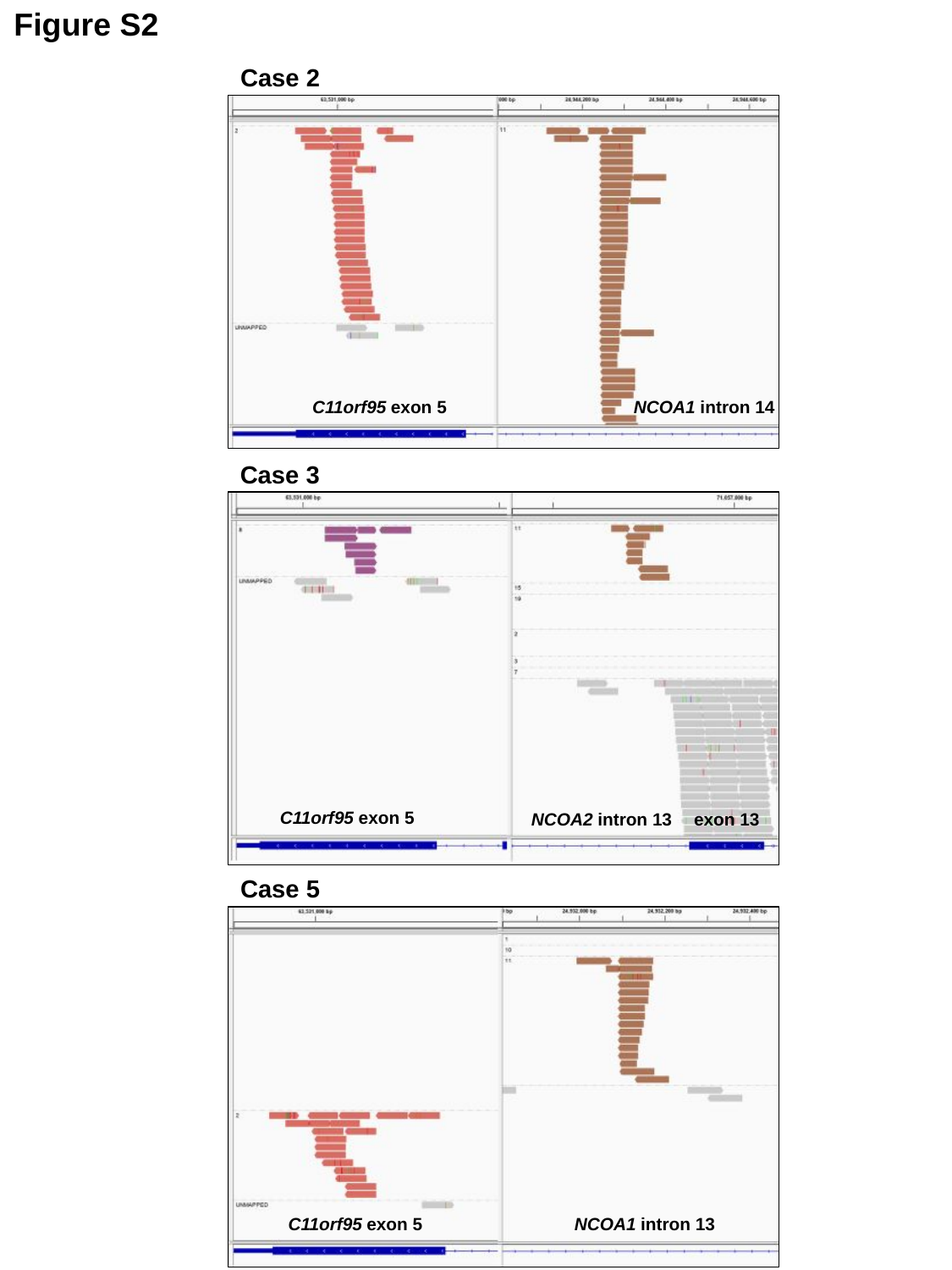

Figure S2
Case 2
C11orf95 exon 5
NCOA1 intron 14
Case 3
C11orf95 exon 5
NCOA2 intron 13
exon 13
Case 5
C11orf95 exon 5
NCOA1 intron 13
