# Supplementary material for: Ependymoma‐like tumor with mesenchymal differentiation harboring C11orf95‐NCOA1/2 or ‐RELA fusion: A hitherto unclassified tumor related to ependymoma
Source: Brain Pathol. 2021 Feb 12;31(3):e12943. doi: 10.1111/bpa.12943 (PMC8412126; doi:10.1111/bpa.12943)
Supplement: Supplementary file 3 — FIGURE S3 (A) In case 4 with C11orf95‐RELA, array comparative genomic hybridization shows no apparent copy number changes in chromosomes 1, 3, 9, 10, 11, or 22, where supratentorial ependymomas with C11orf95‐RELA were reported to have abundant copy number changes. (B) Copy number analysis using the DKFZ methylation classifier demonstrated stable chromosomal status with no apparent copy number changes in cases 3 and 5 [file BPA-31-e12943-s005.pptx]

## Slide 1
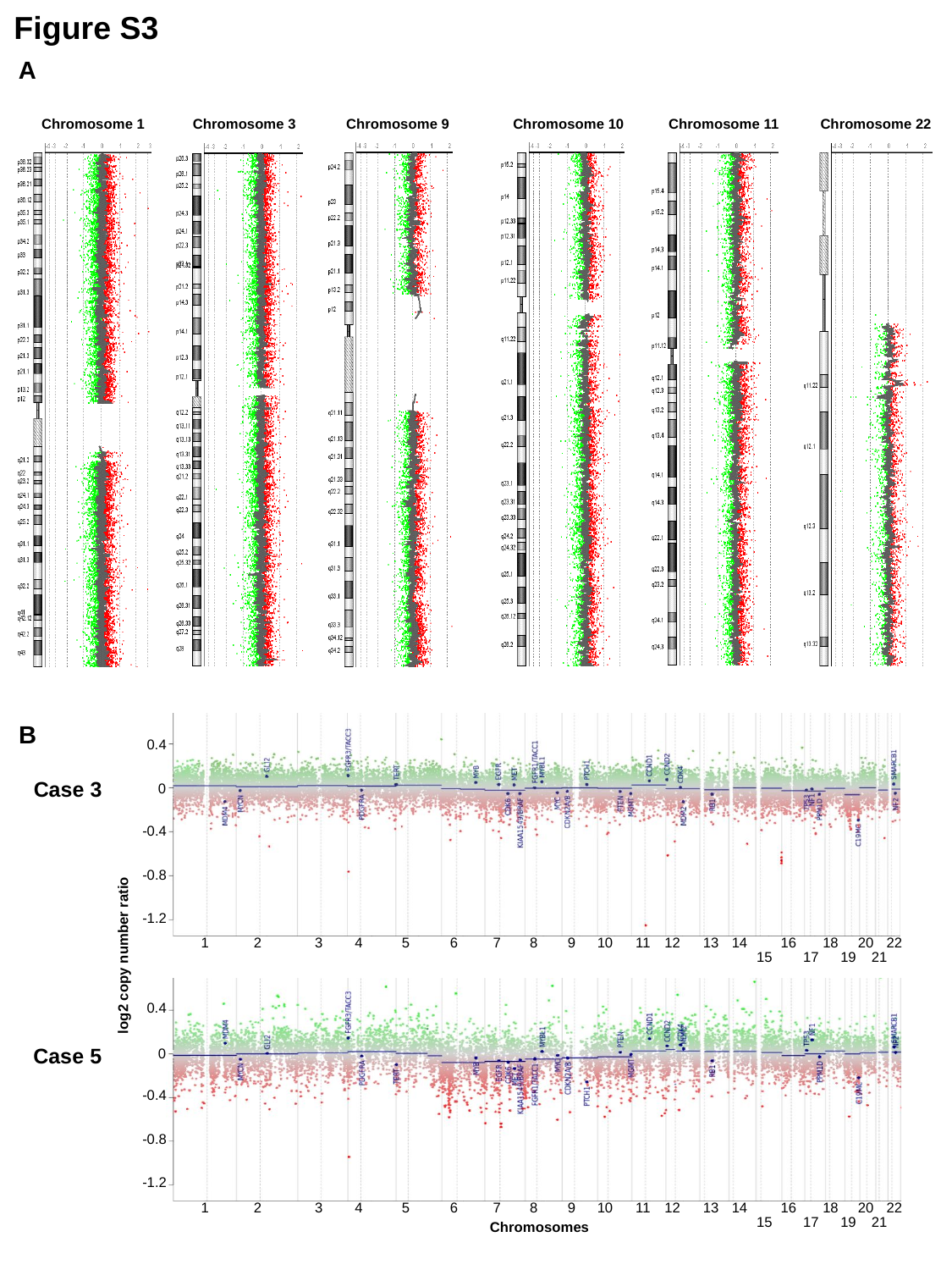

Figure S3
A
Chromosome 22
Chromosome 11
Chromosome 10
Chromosome 9
Chromosome 3
Chromosome 1
B
0.4
Case 3
0
-0.4
-0.8
-1.2
18
9
10
7
3
4
1
11
13
22
5
16
20
8
12
14
2
6
log2 copy number ratio
17
15
21
19
0.4
Case 5
0
-0.4
-0.8
-1.2
18
9
10
7
3
4
1
11
13
22
5
16
20
8
12
14
2
6
17
15
21
19
Chromosomes
