# Supplementary material for: Ependymoma‐like tumor with mesenchymal differentiation harboring C11orf95‐NCOA1/2 or ‐RELA fusion: A hitherto unclassified tumor related to ependymoma
Source: Brain Pathol. 2021 Feb 12;31(3):e12943. doi: 10.1111/bpa.12943 (PMC8412126; doi:10.1111/bpa.12943)
Supplement: Supplementary file 4 — Supplementary Material [file BPA-31-e12943-s004.docx]

**Supplementary Methods**

RNA sequencing and reverse transcriptase-polymerase chain reaction (RT-PCR)

Total RNA was extracted from FFPE (cases 2 and 5) or frozen (case 3) samples. RNA sequencing libraries were prepared using a TruSight RNA Pan-Cancer library kit (Illumina, San Diego, CA, USA) from the FFPE samples and a SureSelect Strand Specific RNA Library Prep Kit (Agilent Technologies, Santa Clara, CA, USA) from the frozen sample. The libraries were subjected to paired-end sequencing of 151-base and 101-base pair fragments on a MiSeq and HiSeq2500 DNA sequencer, respectively (Illumina, Sandiego, CA, USA) according to the manufacturer’s instructions. We obtained more than 70 million reads from each sample, and the paired-end reads were mapped and aligned to known RNA sequences in the RefSeq, Ensembl, and LincRNA databases, and gene fusions were detected using our in-house pipeline as previously described [8]. For RT-PCR analysis, total RNA was reverse-transcribed into complementary DNA with iScript (Bio-Rad, Hercules, CA, USA). The cDNA was subjected to PCR amplification with GXL-Taq (Takara Bio, Tokyo, Japan). PCR primers used for fusion detection were as follows; 5’-CCA TCA AGC GCC ACA TCC T-3’ for exon 5 of *C11orf95*, 5’-ATC TCC GAT TTG ATG GTT ACA CT-3’ for exon 12 of *NCOA1*.

Whole exome sequencing (WES)

WES was performed on DNA of sufficient quality and quantity isolated from FFPE tissue of cases 2-5 using a NextSeq 500 DNA sequencer, as described previously [2], with the mean coverage depths for cases 2-5 being 98, 293.4, 482.6, and 55-fold, respectively. The percentage of target coverage at > 20x was 96.2, 99.2, 99.3, and 90.3 for cases 2-5, respectively. Variants were annotated using the ANNOVAR database (http://annovar.openbioinformatics.org/en/latest/). Exonic and splicing-site variants were selected for analysis, and those represented in dbSNP Build 132 (https://www.ncbi.nlm.nih.gov/projects/SNP/) and synonymous variations were filtered out. The remaining variants were evaluated for their putative functional importance using PolyPhen2 [1] and those with benign predictions were filtered out. Variants observed in more than 2 cases were selected. Furthermore, by manual inspection of aligned reads using Integrative Genomics Viewer (http://www.broadinstitute.org/igv/), multiple variants detected in single reads were removed probably due to mapping errors. We browsed the Catalogue Of Somatic Mutations In Cancer (COSMIC) v70 (https://cancer.sanger.ac.uk/cosmic/) and ClinVar (https://www.ncbi.nlm.nih.gov/clinvar/) to clarify whether the variants detected were previously reported to be associated with diseases. Selected variants were categorized as follows: COSMIC database-registered variants, truncation mutation (not registered in COSMIC database) or variants of unknown significance (VUS) (Supplementary Table 2).

Genome-wide DNA methylation analysis

DNA of sufficient quality and quantity was extracted from cases 3 (frozen sample) and 5 (FFPE sample), and bisulfite modification of DNA was performed using an EZ Methylation DNA Kit (Zymo Research, CA, USA). Methylation profiling was performed using an Infinium Methylation EPIC BeadChip array (Illumina, San Diego, CA, USA), which includes 866,238 CpG sites. For the EPIC array data, we removed 57,277 failed probes (detected p-value > 0.05), 16,257 probes on the sex chromosomes, and 26,353 probes with SNPs at CpG sites. A total of 24,267 cross-reactive probes were also excluded [5]. Remaining probes for analysis numbered 742,084. The methylation level of each CpG site was described by beta-values, which ranged from zero (unmethylated) to one (fully methylated). Preprocessing for analysis was performed with the minfi package [3,6] using R software, version 3.4.1. The data were normalized using the ssNoob method, which is part of the minfi package [3]. The ComplexHeatmap package [7] was used for unsupervised hierarchical clustering on Pearson distance with a Ward algorithm. The 5000 highest median absolute deviation (MAD) probes on CpG islands were selected for analysis. Methylation profiles were analyzed with t-distributed stochastic neighbor embedding (t-SNE) in two dimensions using the Rtsne package (https://github.com/jkrijthe/Rtsne). A Pearson correlation matrix of the 5000 probes was used to generate t-SNE plots, with a theta value of zero over 10,000 iterations. The reference methylation data of 380 central nervous system tumors (GSE90496) were obtained from the Gene Expression Omnibus database (http://www.ncbi.nlm.nih.gov/geo/) for comparison with our data [4]. A molecular classifying algorithm from the German Cancer Center (DKFZ classifier, https://www.molecularneuropathology.org/mnp) was also performed. The classifier designates molecular “class” based on comparison between observed methylation data and a reference cohort consisting of over 2800 neuropathological tumors of almost all known entities [4]. The DNA copy number was calculated from raw signal intensities from the methylation data using the Conumee package (http://bioconductor.org/packages/conumee/) and also obtained from the webpage of German Cancer Center as described above.

Array comparative genomic hybridization (CGH)

DNA of sufficient quality and quantity extracted from FFPE samples of cases 2-5 was analyzed by array CGH. Array CGH analysis was carried out using a 4×180K CGH oligonucleotide microarray (Agilent Technologies, Santa Clara, CA, USA), as described previously [9]. The sizes of gains and losses were refined by manual inspection of probe intensity plots. The log2 ratio of <-1.0 at the region of interest was considered to represent a homozygous deletion, and a value of -1.0 to -0.2 was considered to represent a heterozygous deletion [10].

**References**

1. Adzhubei IA, Schmidt S, Peshkin L, Ramensky VE, Gerasimova A, Bork P, Kondrashov AS, Sunyaev SR (2010) A method and server for predicting damaging missense mutations. Nat Methods 7:248-249.

2. Arai H, Nobusawa S, Kawabata-Iwakawa R, Rokudai S, Higuchi T, Yamazaki T, Horiguchi J, Sano T, Kojima M, Nishiyama M, Yokoo H, Hirato J, Oyama T (2018) Myeloid sarcoma arising in malignant phyllodes tumour: clonal relationships revealed by comparative genome-wide analyses. Br J Haematol 181:255-259.

3. Aryee MJ, Jaffe AE, Corrada-Bravo H, Ladd-Acosta C, Feinberg AP, Hansen KD, Irizarry RA (2014) Minfi: a flexible and comprehensive Bioconductor package for the analysis of Infinium DNA methylation microarrays. Bioinformatics 30:1363-1369.

4. Capper D, Jones DTW, Sill M, Hovestadt V, Schrimpf D, Sturm D, Koelsche C, Sahm F, Chavez L, Reuss DE, Kratz A, Wefers AK, Huang K, Pajtler KW, Schweizer L, Stichel D, Olar A, Engel NW, Lindenberg K, Harter PN, Braczynski AK, Plate KH, Dohmen H, Garvalov BK, Coras R, Hölsken A, Hewer E, Bewerunge-Hudler M, Schick M, Fischer R, Beschorner R, Schittenhelm J, Staszewski O, Wani K, Varlet P, Pages M, Temming P, Lohmann D, Selt F, Witt H, Milde T, Witt O, Aronica E, Giangaspero F, Rushing E, Scheurlen W, Geisenberger C, Rodriguez FJ, Becker A, Preusser M, Haberler C, Bjerkvig R, Cryan J, Farrell M, Deckert M, Hench J, Frank S, Serrano J, Kannan K, Tsirigos A, Brück W, Hofer S, Brehmer S, Seiz-Rosenhagen M, Hänggi D, Hans V, Rozsnoki S, Hansford JR, Kohlhof P, Kristensen BW, Lechner M, Lopes B, Mawrin C, Ketter R, Kulozik A, Khatib Z, Heppner F, Koch A, Jouvet A, Keohane C, Mühleisen H, Mueller W, Pohl U, Prinz M, Benner A, Zapatka M, Gottardo NG, Driever PH, Kramm CM, Müller HL, Rutkowski S, von Hoff K, Frühwald MC, Gnekow A, Fleischhack G, Tippelt S, Calaminus G, Monoranu CM, Perry A, Jones C, Jacques TS, Radlwimmer B, Gessi M, Pietsch T, Schramm J, Schackert G, Westphal M, Reifenberger G, Wesseling P, Weller M, Collins VP, Blümcke I, Bendszus M, Debus J, Huang A, Jabado N, Northcott PA, Paulus W, Gajjar A, Robinson GW, Taylor MD, Jaunmuktane Z, Ryzhova M, Platten M, Unterberg A, Wick W, Karajannis MA, Mittelbronn M, Acker T, Hartmann C, Aldape K, Schüller U, Buslei R, Lichter P, Kool M, Herold-Mende C, Ellison DW, Hasselblatt M, Snuderl M, Brandner S, Korshunov A, von Deimling A, Pfister SM (2018) DNA methylation-based classification of central nervous system tumours. Nature 555:469-474.

5. Chen YA, Lemire M, Choufani S, Butcher DT, Grafodatskaya D, Zanke BW, Gallinger S, Hudson TJ, Weksberg R (2013) Discovery of cross-reactive probes and polymorphic CpGs in the Illumina Infinium HumanMethylation450 microarray. Epigenetics 8:203-209.

6. Fortin JP, Triche TJ Jr, Hansen KD (2017) Preprocessing, normalization and integration of the Illumina HumanMethylationEPIC array with minfi. Bioinformatics 33:558-560.

7. Gu Z, Eils R, Schlesner M (2016) Complex heatmaps reveal patterns and correlations in multidimensional genomic data. Bioinformatics 32:2847-2849.

8. Nakamura H, Arai Y, Totoki Y, Shirota T, Elzawahry A, Kato M, Hama N, Hosoda F, Urushidate T, Ohashi S, Hiraoka N, Ojima H, Shimada K, Okusaka T, Kosuge T, Miyagawa S, Shibata T (2015) Genomic spectra of biliary tract cancer. Nat Genet 47:1003-1010.

9. Nobusawa S, Lachuer J, Wierinckx A, Kim YH, Huang J, Legras C, Kleihues P, Ohgaki H (2010) Intratumoral patterns of genomic imbalance in glioblastomas. Brain Pathol 20:936-944.

10. Tagawa H, Karnan S, Suzuki R, Matsuo K, Zhang X, Ota A, Morishima Y, Nakamura S, Seto M (2005) Genome-wide array-based CGH for mantle cell lymphoma: identification of homozygous deletions of the proapoptotic gene BIM. Oncogene 24:1348-1358.
